# Relationship of left ventricular non-compaction with papillary muscle insertion site and partition

**DOI:** 10.1186/1532-429X-11-S1-P239

**Published:** 2009-01-28

**Authors:** Mitra Sahebazamani, Ijaz Ahmad, Geetha P Bhumireddy, Igor Klem, Joshua A Socolow, Terrence Sacchi, John F Heitner

**Affiliations:** 1 New York Methodist Hospital, Brooklyn, NY USA; 2 Duke Univ Medical Center, Durham, NC USA

**Keywords:** Left Ventricle, Cardiac Magnetic Resonance, Insertion Site, Papillary Muscle, Ventricular Apex

## Introduction

Left ventricular non-compaction (LVNC) is a rare congenital morphogenetic abnormality and occurs as a result of an arrest in the compaction of the embryonic myocardium during development resulting in excessive trabeculations in the left ventricle (LV). The relationship of the papillary muscle (PM) development and LVNC has not been previously described.

## Purpose

To assess the correlation between LVNC and both the degree of partitions as well as the insertion site (apical vs. mid-ventricular) of PM as compared to patients with normal compacted myocardium.

## Methods

We enrolled 297 consecutive patients referred to our cardiac magnetic resonance (CMR) center for cardiac evaluation. We assessed the non-compacted (trabecular region) and compacted myocardium by drawing diameters of each at the left ventricular apex and averaging from two long axis cine views. LVNC was defined as non-compacted to compacted ratio 2.3. The PM insertion site was determined by dividing the long axis of the LV into 3 equal regions and determining in which region (mid or apical) the papillary muscle inserted into the LV. The number of partitions of the PM was determined by counting the number of separate PM visualized 10 mm apical from the mid plane of the LV on short axis cine view.

## Results

The mean age of the patients was 58.1 years, 60% were males. The patients were referred to CMR for the following reasons: evaluation of left ventricular function (37%), viability (30%), valvular diseases (21%), and other (12%). The average LVEF was 51.2 ± 14.6%. The mean number of PM partition in the LVNC group was 5, and in the normal group was 6 (p = 0.41). The LVNC group had a significantly higher apical insertion of the PM compared to the normal group, 87% (67 of 77 patients) vs 67.3% (148 of 220 patients, p < 0.001).

## Conclusion

Patients with LVNC have a higher incidence of apical insertion of the papillary muscle into the myocardium, without a higher number of partitions.

